# Comparison of progesterone-modified natural cycle and hormone replacement therapy cycle for endometrial preparation in single frozen blastocyst transfer (COMPROSET): protocol for an open-label randomized controlled trial

**DOI:** 10.3389/fmed.2025.1522004

**Published:** 2025-04-28

**Authors:** Hai-Ning Yuan, Jing-Yan Song, Zhen-Gao Sun

**Affiliations:** ^1^The First Clinical College, Shandong University of Traditional Chinese Medicine, Jinan, China; ^2^Reproductive Center of Integrated Medicine, The Affiliated Hospital of Shandong University of Traditional Chinese Medicine, Jinan, China

**Keywords:** progesterone-modified natural cycle, frozen–thawed embryo transfer, live birth, randomized controlled trial, hormone replacement therapy cycle

## Abstract

**Introduction:**

As infertility incidence increases gradually, frozen–thawed embryo transfer (FET) techniques are continuously being developed and enhanced. Although endometrial preparation regimens are strongly linked to live birth rates (LBR), there has been controversy over the ideal regimen. Hormone replacement therapy (HRT) cycles, despite the ease of synchronizing embryo thawing and embryo transfer timing, increase the risk of pregnancies and obstetric complications compared to natural cycles (NC). By ensuring the presence of the corpus luteum while reducing the number of monitoring sessions, the progesterone modified natural cycle (P_4_mNC) offers more convenience for the patient than the normal NC. This study is designed to compare the effects of P4mNC and HRT cycles on FET outcomes.

**Methods and analysis:**

This study is a single-center, open-label, randomized controlled trial (RCT) targeting to recruit a total of 672 women, with 336 individuals each in two arms (1:1 treatment ratio). Women undergoing in vitro fertilization (IVF) scheduled for a single frozen–thawed blastocyst transfer (FBT) and who have regular menstrual cycles are eligible. After signing an informed consent form, patients will be randomized into two groups: the P4mNC group and the HRT group. The primary objective is to determine whether P_4_mNC-FBT is non-inferior to standard HRT-FBT in terms of LBR. Intention-to-treat analysis will be used for data analysis.

**Ethics and dissemination:**

This study is approved by the Institutional Review Board of the Affiliated Hospital of Shandong University of Traditional Chinese Medicine (Reference No. 2024-144-KY). The results of our study will be submitted to reproductive medicine conferences and journals.

**Clinical trial registration:**

ClinicalTrials.gov, identifier NCT06644794.

## Highlights

- This is the first and largest randomised controlled trial to date to examine the clinical outcomes between P_4_mNC and HRT for FBT.
- This is a RCT that focuses primarily on LBR, but also assesses maternal and neonatal outcomes as well.
- This is an open-label trial and only targets infertile women with regular ovulation.

## Introduction

A global population survey estimates that 186 million people suffer from infertility (1). As couples postpone childbearing, infertility incidence will gradually increase (2). Thus, infertility poses a persistent reproductive problem worldwide (1). As a result, this has driven advances and improvements in assisted reproductive technology (ART) (3). As vitrification freezing has been introduced and reliable safety data have been validated, the proportion of FET cycles has increased (4–7). There are currently several protocols for endometrial preparation, including true natural cycle (NC) with spontaneous ovulation, modified natural cycle (mNC) with human chorionic gonadotropin (hCG)-triggered ovulation, ovarian stimulation cycles, and hormone replacement therapy (HRT) cycles with or without gonadotropin-releasing hormone agonist (GnRH-a) downregulation (8). Nevertheless, the topic of the optimal protocol for endometrial preparation continues to be controversial (9, 10).

During HRT cycles, embryo thawing and transfer can be coordinated easily. However, recent findings suggest pregnancies resulting from anovulation without the corpus luteum (CL) in HRT cycles may pose potential risks, including an increased likelihood of preeclampsia, preterm birth, and low birth weight, to the mother and fetus compared to NC (11, 12). These differences are probably due to the lack of vasoactive substances produced by the CL (13, 14). As a result, the NC protocol is simpler and more physiologic, and it is more commonly used for endometrial preparation for FET in patients with regular menstruation and normal ovulation (15). However, NC-FET has the disadvantage of being less flexible as it requires more frequent clinical visits to closely monitor follicular development, endometrial thickness, and hormone levels (16). In contrast, mNC is very similar to NC, but ovulation is triggered by hCG, which allows for less frequent monitoring and more convenience for patients, but still requires regular monitoring with ultrasound and serum luteinizing hormone (LH) levels (16, 17). New protocols for endometrial preparation are crucial to enhance the flexibility of FET planning while maintaining ovulation and the presence of the CL. Recently, a retrospective cohort study showed that no inferiority of the P_4_mNC-FBT cycle was detected in terms of clinical pregnancy rate, miscarriage rate, and LBR compared with the HRT-FBT cycle (18). Therefore, we designed this randomized controlled trial (RCT) to further validate the efficacy and safety of the P_4_mNC as endometrial preparation protocol in FBT.

## Objectives

### Primary objective

The primary objective of the study is to investigate if P_4_mNC-FBT is non-inferior to standard HRT-FBT in terms of LBR. ITT and per-protocol (PP) analyses will be performed with a non-inferiority margin of 5%. A live birth is defined as the delivery of any surviving newborn at 28 weeks or more of gestation.

### Secondary objective

1. Biochemical pregnancy: defined as serum level of ß-hCG > 50 mIU/mL.
2. Clinical pregnancy: defined as fetal heartbeat observed by vaginal ultrasound at 5 weeks after embryo transfer.
3. Ongoing pregnancy: defined as the presence of a gestational sac and fetal heartbeat after 12 weeks of gestation.
4. Miscarriage is defined as a condition in which the embryo or fetus does not survive and is not spontaneously absorbed or expelled from the uterus.
5. Pregnancy complications include ectopic pregnancy, hyperemesis gravidarum, hypertensive disorders of pregnancy, gestational diabetes mellitus, preterm birth.
6. Birth weight, including low birth weight (defined as weight < 2,500 g at birth), very low birth weight (defined as <1,500 g at birth), high birth weight (defined as >4,000 g at birth) and very high birth weight (defined as >4,500 g at birth).
7. Large for gestational age (defined as birth weight > 90th centile for gestation, based on standardized ethnicity-based charts) and small for gestational age (defined as < 10th centile for gestational age at delivery based on standardized ethnicity-based charts).
8. Birth defects are identifiable structural or functional defects that develop in the fetus in utero at birth.

## Methods and analysis

### Study design

**Figure 1:**
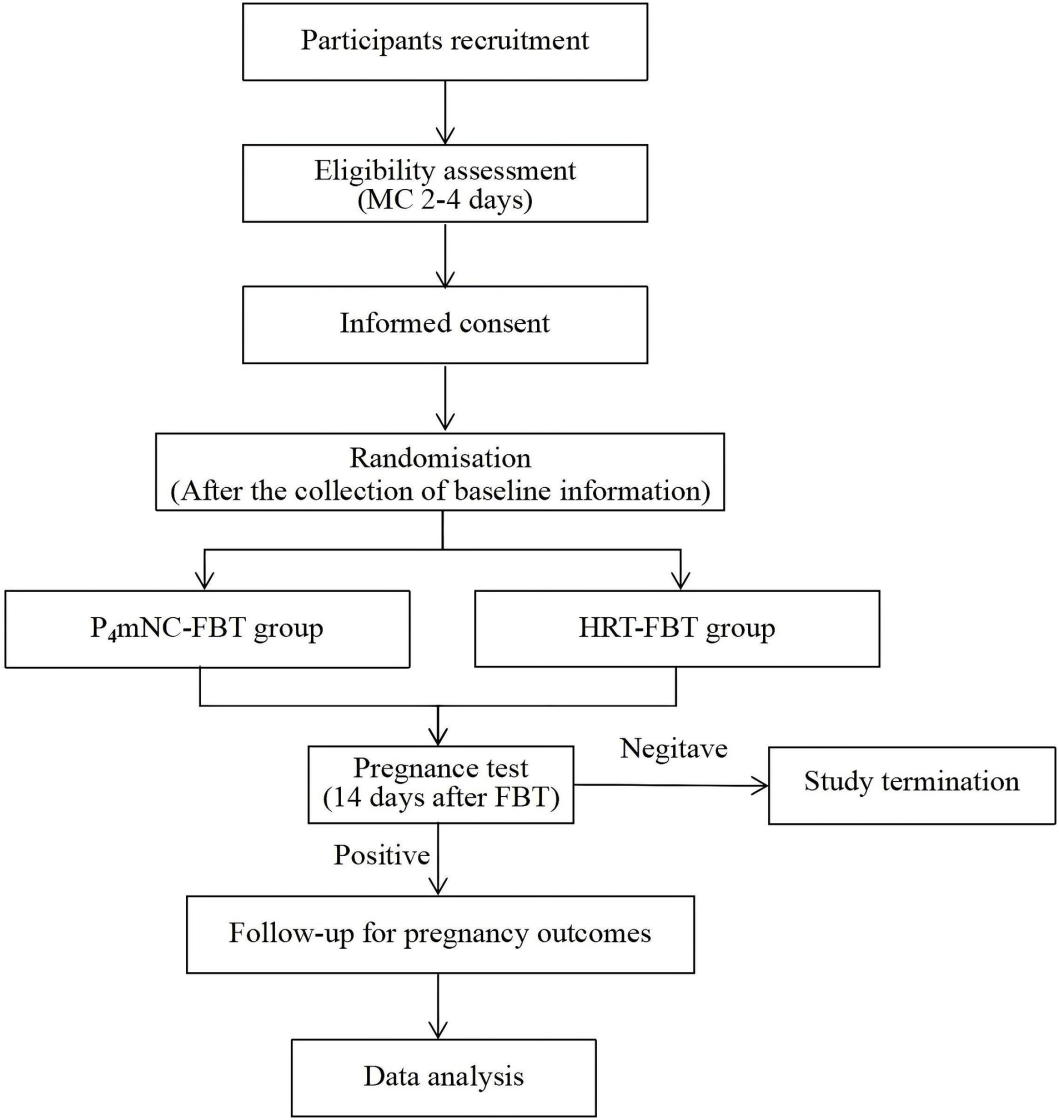
The study flow chart. MC, the menstrual cycle; P_4_mNC, progesterone modified natural cycle; FBT, frozen–thawed blastocyst transfer. HRT, hormone replacement therapy.

This study protocol describes the design of a single-center, open-label, randomized, controlled trial. Patients will be recruited from the reproductive center of the Affiliated Hospital of Shandong University of Traditional Chinese Medicine (SDUTCM). To ensure procedural consistency, all participating investigators will receive uniform training and have regular communication. The study is registered at ClinicalTrials.gov (ID: NCT06644794) in 15 October 2024. Patient enrollment will begin in December 2024 and is scheduled to continue until June 2027. Patients will be randomly assigned to P_4_mNC cycles and HRT cycles. This study has been approved by the Institutional Review Board of Affiliated Hospital of SDUTCM (Reference No. 2024-144-KY). Informed consent will be obtained from each patient before any study procedure (Figure 1).

### Patient and public involvement

**Figure 2:**
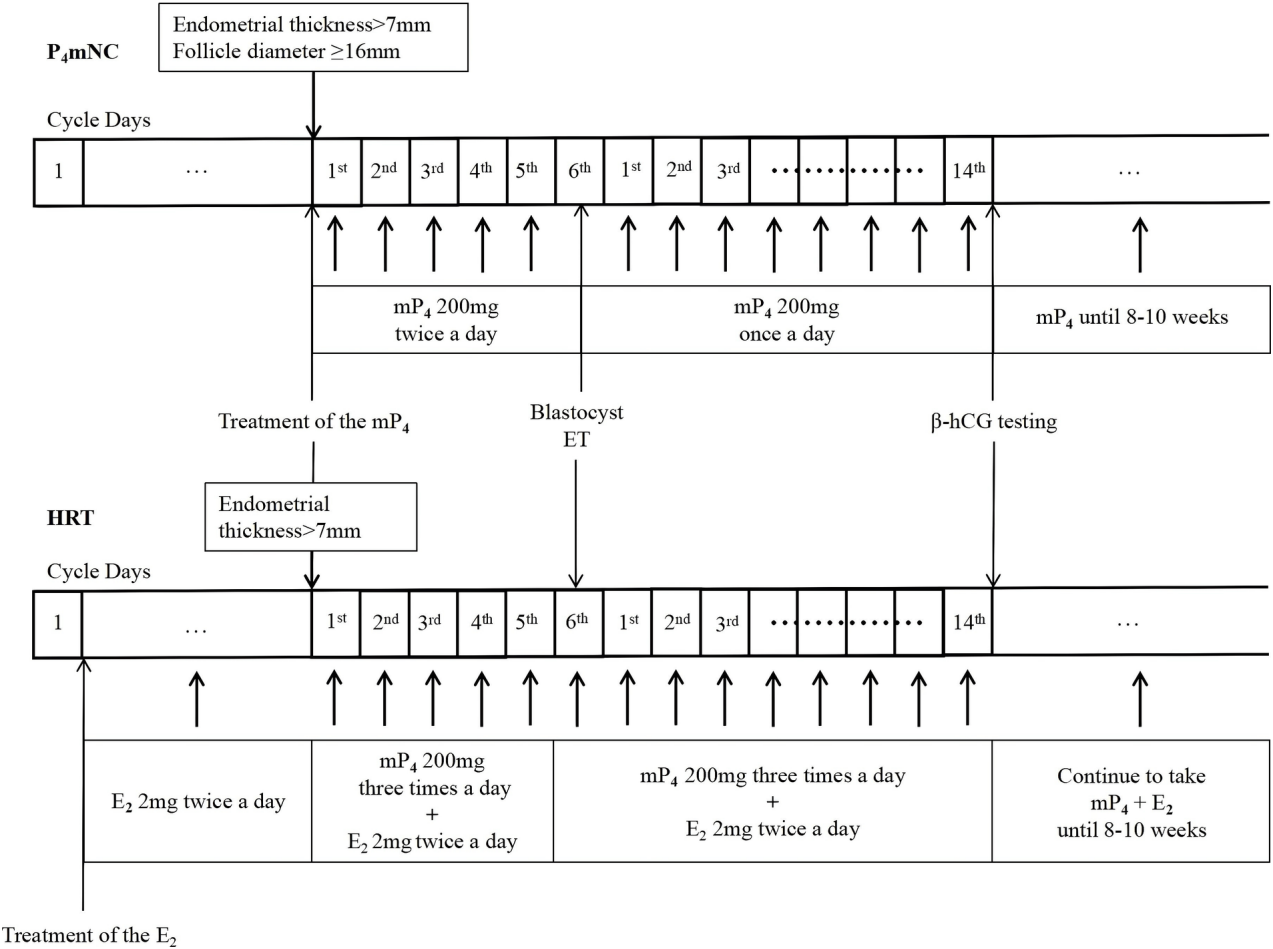
Scheme of endometrial preparation in progesterone modified natural and hormone replacement therapy cycles. ET, embryo transfer; mP4, micronized progesterone; *β*-hCG, beta human chorionic gonadotropin; P4mNC, progesterone modified natural cycle; HRT, hormone replacement therapy.

Lack of patient and public involvement in the formulation of research questions, study design or recruitment, and study conduct and reporting (Figure 2).

### Study population and recruitment

A total of 672 women undergoing autologous single FBT at the reproductive center of the Affiliated Hospital of SDUTCM will be recruited. Prior to initiating treatment, investigators will browse the files of patients scheduled for FET and screen them including uterine and ovarian ultrasound on day 2 or 4 of their menstrual cycle to ensure that enrollment criteria are met. Details about the trial will be presented at their first visit. After obtaining approval from the ethics committee, all women will provide written informed consent prior to participation. At any time during the study, the patient can withdraw without reason.

### Eligibility criteria

#### Inclusion criteria

- Patients aged 21 to 44 years undergoing FBT;
- Body mass index (BMI) 18–35 kg/m^2^;
- Having regular ovulatory cycles.

#### Exclusion criteria

- Untreated uterine adhesions;
- Medical contraindications to estrogen and progesterone therapy;
- Illnesses contraindicating assisted reproductive technology or pregnancy;
- History of recurrent implantation failures (> 2 embryo transfer failures);

### Treatment and interventions

Patients will undergo ovarian stimulation and oocyte retrieval according to the protocol of the attending physician. Final oocyte maturation will be induced using recombinant hCG (250 μg, Ovitrelle, Serono, France) or GnRH agonist (0.1 mg, Triptoreline, Decapeptyl, Ipsen, France). After 35–36 h, oocyte retrieval is performed transvaginally under ultrasound guidance. Within 2–6 h, all retrieved oocytes will be fertilized by either IVF or intracytoplasmic sperm injection (ICSI), and all embryos will be cultured to blastocyst stage. Based on the Gardner classification, a morphological quality score will be assigned to blastocysts. We divide embryos into two groups: blastocysts of good quality with an ICM/TE score type AA, AB, BA, or BB; and poor-quality blastocysts with an ICM/TE score AC or BC. Vitrified-warmed embryos will be transferred in one of two types of cycles: P_4_mNC or HRT.

### P_4_mNC-FBT group

On days 8–12 of the menstrual cycle (MC), depending on the length of the patient’s MC, transvaginal ultrasound is used to monitor follicular development and endometrial growth. Vaginal micronized progesterone (Utrogestan, Besins, Belgium) is started at 200 mg in the afternoon and 200 mg in the evening when the dominant follicle reached ≥16 mm and the endometrial thickness is at least 7 mm. A blastocyst is transferred on day 5 after the addition of progesterone. On day 14 after blastocyst transfer, serum *β*-hCG levels are measured. Upon positive serum pregnancy testing, progesterone support will continue until 8–10 weeks of gestation. However, afternoon progesterone use is eliminated for 30 days after embryo transfer.

### HRT-FBT group

Endometrial preparation will begin on the second day of the menstrual cycle with oral estradiol (E_2_) valerate at a dose of 2 mg twice daily. When the patient’s endometrial thickness is ≥7 mm, vaginal progesterone administration will be initiated at a dose of 200 mg 3 times daily. On day 5 of the progesterone administration, blastocysts are thawed and transferred. For patients with endometrial thickness <7 mm, patients continued oral E_2_ until the endometrium is ≥7 mm. On day 14 after blastocyst transfer, serum *β*-hCG levels are measured. Upon positive serum pregnancy testing, E_2_ and progesterone supplementation is continued for 8–10 weeks of gestation.

### Randomization and blinding

Statistical professionals not involved in the study will complete the randomization groupings using software (R 4.0.0, R Foundation for Statistical Computing, Vienna, Austria), and stratify by female age (<35 or ≥35 years). Patients will then be randomized in a 1:1 ratio to the P_4_mNC-FBT and HRT-FBT groups. Both the investigators and patients will be aware of the allocation. Blinding of the intervention is impossible for patient and doctors administering it. To minimize performance and reporting biases arising from the absence of blinding, embryologists and doctors involved in embryo transfer are blinded to the group assignments of the participants. Subjective outcomes, such as pregnancy complications, were assessed by strictly adhering to internationally recognized diagnostic guidelines to ensure objectivity. Additionally, patients received standardized instructions for symptom reporting to avoid discrepancies in self-described experiences.

### Data collection and management

Relevant treatment data for patients will be collected at the appropriate time points: (1) baseline (MC 2–4 days); (2) day of blastocyst transfer (same day); and (3) *β*-hCG (14 days after blastocyst transfer). Follow-up data for all pregnancies resulting from FET will begin with inclusion in the study and continue for 1 year, according to the study protocol. Based on the anonymous subject ID numbers used in the trial, the database has a complete audit trail. Participant information sheets will be prepared to ensure data accuracy and regular data monitoring and validation, and data will be backed up once a day (on another computer in the same physical location as the server).

## Statistics

### Non-inferiority design and power calculation

A non-inferiority design is used as the new treatment, P_4_mNC-FBT, is expected to yield comparable results, but will offer significant advantages over the current standard treatment (HRT-FBT) in terms of patient convenience, reduced incidence of gestational hypertensive disorders, and lower costs as the progesterone dosage is reduced. In a previous study, the LBR is 45% in the P_4_mNC-FBT group and 39.6% in the HRT-FBT group (18). Sample sizes of 302 in P_4_mNC group and 302 in HRT group achieve 80.069% power to detect a difference of 0.05 when the non-inferiority difference is −0.05. The reference group proportion is 0.4. The treatment group proportion is assumed to be 0.35 under the null hypothesis. The power was computed for the case when the actual treatment group proportion is 0.45. The test statistic used is the one-sided Z test (unpooled). The significance level of the test is 0.05. A total of 672 patients are finally included in both groups, taking into account a 10% dropout rate.

### Statistical analysis

ITT analyses include drop-outs and canceled cycles, PP analyses include canceled cycles but not drop-outs, and per-transfer analyses exclude both drop-outs and canceled cycles. Differences in LBR will be evaluated by means of risk differences with one-sided 95% CI (or equivalently two-sided 90% CI). Non-inferiority will be concluded if the CI excludes a difference of more than 5% in favor of the present standard treatment (HRT-FBT) in ITT and PP analyses. Depending on the characteristics of the baseline data, continuous data will be expressed as mean ± SD or median and interquartile spacing (IQR). Categorical data will be described by frequencies and percentages. The primary outcome, LBR, will be compared between the two groups using the *χ*^2^ test as well as risk ratios and 95% CI. Quantitative variables will be analyzed using the student *t*-test or Mann–Whitney test. Secondary outcomes will be compared between the two arms using the similar approach described for the primary outcome. Data will be analyzed using SPSS 26.0 and a *p*-value <0.05 will be considered statistically significant. Any missing data will be handled using pairwise deletion.

## Ethics and dissemination

The study was approved by the Institutional Review Board of the Affiliated Hospital of SDUTCM (No. 2024-144-KY). Written informed consent is obtained by the investigators from each patient participating in the study prior to the start of the study. The safety of the subjects is high because the pharmacological treatments involved in the P_4_mNC-FET regimen and the HRT-FET regimen are well recognized. The Independent Data Safety Monitoring Board (DSMB) conducted interim safety analyses at predefined enrollment milestones. Serious adverse events (SAEs) were reported to the ethics committee and DSMB within 48 h of occurrence. In case of serious adverse events or suspected and unanticipated serious adverse reactions in subjects the trial will be stopped immediately for treatment. In addition, they are free to withdraw from the study at any time without giving any reason. Data will be entered electronically and all data will be stored in locked computer files accessible only to the participating investigators and study staff and will be kept for 3 years after completion of the study. The investigators always maintain a strict privacy policy. The results of our study will be presented to reproductive medicine conferences and journals.
